# The Role and Applications of Artificial Intelligence in Dental Implant Planning: A Systematic Review

**DOI:** 10.3390/bioengineering11080778

**Published:** 2024-07-31

**Authors:** Monica Macrì, Vincenzo D’Albis, Giuseppe D’Albis, Marta Forte, Saverio Capodiferro, Gianfranco Favia, Abdulrahman Omar Alrashadah, Victor Diaz-Flores García, Felice Festa

**Affiliations:** 1 Department of Innovative Technologies in Medicine & Dentistry, University “G. D’Annunzio” of Chieti-Pescara, 66100 Chieti, Italy; vincenzo.dalbis@studenti.unich.it (V.D.); ffesta@unich.it (F.F.); 2 Department of Interdisciplinary Medicine, University of Bari Aldo Moro, 70121 Bari, Italy; giuseppe.dalbis@uniba.it (G.D.); marta.forte@uniba.it (M.F.); saverio.capodiferro@uniba.it (S.C.); gianfranco.favia@uniba.it (G.F.); 3 Department of Dentistry, King Faisal University, P.O. Box 380, Al Hofuf 31982, Saudi Arabia; dr.alrashadah@gmail.com; 4 Department of Pre-Clinical Dentistry, School of Biomedical Sciences, Universidad Europea de Madrid, Villaviciosa de Odón, 28670 Madrid, Spain; victor.diaz-flores@universidadeuropea.es

**Keywords:** artificial intelligence in implantology, AI in dental implantology, machine learning in dental implantology, deep learning in implant dentistry, robotics in dental implantology, AI-based implant planning

## Abstract

Artificial intelligence (AI) is revolutionizing dentistry, offering new opportunities to improve the precision and efficiency of implantology. This literature review aims to evaluate the current evidence on the use of AI in implant planning assessment. The analysis was conducted through PubMed and Scopus search engines, using a combination of relevant keywords, including “artificial intelligence implantology”, “AI implant planning”, “AI dental implant”, and “implantology artificial intelligence”. Selected articles were carefully reviewed to identify studies reporting data on the effectiveness of AI in implant planning. The results of the literature review indicate a growing interest in the application of AI in implant planning, with evidence suggesting an improvement in precision and predictability compared to traditional methods. The summary of the obtained findings by the included studies represents the latest AI developments in implant planning, demonstrating its application for the automated detection of bones, the maxillary sinus, neuronal structure, and teeth. However, some disadvantages were also identified, including the need for high-quality training data and the lack of standardization in protocols. In conclusion, the use of AI in implant planning presents promising prospects for improving clinical outcomes and optimizing patient management. However, further research is needed to fully understand its potential and address the challenges associated with its implementation in clinical practice.

## 1. Introduction

Artificial intelligence (AI) has become increasingly prevalent in various fields of medicine, revolutionizing healthcare delivery and patient outcomes. From diagnostic imaging to treatment planning, AI technologies have demonstrated remarkable capabilities in enhancing clinical decision-making and improving efficiency [1]. In dentistry, AI is emerging as a valuable tool for enhancing various aspects of patient care, including implant planning—a critical component of dental implantology that demands precision and meticulous planning. By leveraging AI algorithms and machine learning techniques, clinicians can analyze complex datasets and optimize treatment strategies for individual patients [2]. Implant planning involves the evaluation of the patient’s anatomy, bone density, and other factors to determine the optimal position, size, and angle of dental implants. Traditionally, this process has relied heavily on the expertise and experience of dental professionals, often involving manual measurements and subjective assessments [3,4].

However, the integration of AI into implant planning brings forth a new era of precision and efficiency. AI algorithms can analyze vast amounts of patient data, including radiographic images, three-dimensional scans, and clinical records, to assist clinicians in making evidence-based decisions regarding implant placement [5]. Moreover, AI can provide predictive modeling and simulation capabilities, allowing clinicians to visualize the expected outcomes of different treatment approaches before initiating the procedure. This not only enhances treatment planning but also enables personalized and patient-specific interventions [6]. Despite the potential benefits, the widespread adoption of AI in implant planning raises several ethical, legal, and practical considerations. Issues such as data privacy, algorithm transparency, and liability pose significant challenges that must be addressed to ensure the responsible and ethical use of AI technologies in dentistry [7].

The aim of this article is to explore the role and applications of AI in implant planning by examining the current state of the art. Additionally, the ethical and legal implications associated with the integration of AI into clinical practice will be discussed, providing insights into the opportunities and challenges of this rapidly evolving field. Through a comprehensive review of the literature, dental professionals will be provided with a deeper understanding of the potential of AI in implant planning and its implications for patient care, clinical workflow, and professional responsibilities.

## 2. Materials and Methods

The final report of this review was prepared in accordance with the guidelines of PRISMA [8] The research question was defined following the Cochrane Manual for Systematic Reviews of the Accuracy of Diagnostic Tests. To address this study question, the PIT (population, index test(s), target condition) methodology was used.

Population (P):

- Description: This systematic review includes studies that utilize AI engines to evaluate 2D and 3D radiological imaging for the diagnostic assessment required for dental implant placement.
- Criteria: Studies must focus on the evaluation of bone quality, bone dimensions, the identification of critical anatomical structures (e.g., nerves and maxillary sinus, adjacent teeth), drilling protocols, and implant position.

Index Test(s) (I):

- Description: The index tests under review are AI-based technologies and tools that assist clinicians in the planning and placement of dental implants. We used AI algorithms that analyze radiological images, provide 3D reconstructions, and suggest optimal implant sites while ensuring the preservation of vital anatomical structures.

Target Condition (T):

- Description: The target condition involves patients with missing teeth (edentulism) who require detailed diagnostic evaluations for the planning of dental implant placement.
- Assessment Focus: The primary focus is on evaluating the AI tools to evaluate the quality and dimensions of the bone and ensuring the safe placement of implants by identifying and preserving critical structures such as nerves and the maxillary sinus.

A comprehensive literature search was conducted on the PubMed, Scopus, and Web of Science databases to identify relevant studies related to the role and applications of artificial intelligence (AI) in implant planning. In addition, Google Scholar was reviewed. 

Further manual exploration of the reference lists of all full-text articles and relevant reviews identified from the electronic search was also conducted. Additionally, manual searches were carried out in the following journals: *Journal of Prosthodontic Research*, *Journal of Prosthetic Dentistry*, *Clinical Oral Implants Research*, *International Journal of Oral Maxillofacial Implants*, *Clinical Implant Dentistry and Related Research*, *Implant Dentistry*, and *Journal of Implantology*. The search was performed using a combination of keywords and Medical Subject Headings (MeSH) terms, including: ((Artificial Intelligence [Mesh] OR (AI) OR (machine learning) OR (deep learning)) AND ((Implant Planning [Mesh] OR (implantology) OR (implant treatment plan)). The inclusion criteria encompassed studies of any level of evidence, excluding expert opinion. Articles with the following study designs were selected: clinical trials, case reports, case series, and in vitro studies. Additionally, only articles published in English and within the period 2020–2024 were considered. An inclusion criterion was that clinical studies be based on human radiological images, excluding those of animals, obtained from the databases of dental clinics of patients undergoing oral implantology. The exclusion criteria were set to omit review articles and letters to editors, as well as animal studies. A summary of the search strategy is described in Table 1. Moreover, studies were excluded if the full text was unavailable. Two independent reviewers screened the titles and abstracts of the retrieved articles to identify potentially relevant studies. Full-text articles were then assessed for eligibility based on the inclusion criteria outlined above. From the selected articles, we gathered the following information: the names of the author(s), the publication year, the country of origin, and the study design. We also noted the total number of patients or datasets, details on the training and validation datasets, and the test datasets used in the studies. 

The article has been registered on INPLASY, the International Platform of Registered Systematic Review and Meta-analysis Protocols. This is the registration number: INPLASY202470123 (https://inplasy.com/inplasy-2024-7-0123/) (accessed on 26 June 2024). The DOI number is 10.37766/inplasy2024.7.0123.

Additionally, we documented the study’s objective, the application of artificial intelligence, and the conclusions and outcomes reported by the authors. The quality of the included studies was evaluated using appropriate assessment tools, specifically the Newcastle–Ottawa Scale (NOS). A star rating system was employed to facilitate a semi-quantitative assessment of the study’s quality. This system, based on the NOS, scores studies from zero to nine stars. Studies were classified as high-quality if they achieved seven or more stars, medium-quality if they received between four and six stars, and poor-quality if they received fewer than four stars (Table 2).

The main characteristics of the included studies, such as the study design, the AI techniques applied, and the published findings, were summarized using descriptive statistics. The research conclusions were examined using a qualitative synthesis, which sought to identify recurring themes, patterns, and areas in which the investigations concurred or differed. Ethical standards and criteria for systematic reviews and meta-analyses were followed in the conduct of this review. Since the study utilized the analysis of publicly available data from other published studies, no ethical approval was needed. The omission of publications written in languages other than English and the potential for publication bias are two potential weaknesses of this review.

Furthermore, the overall conclusions and interpretations could have been impacted by the caliber and diversity of the included studies. Overall, the approach used in this study was to methodically locate, assess, and compile the existing data regarding the function and uses of AI in implant planning, offering insightful information about the state of the field at the moment and guiding future avenues for investigation.

## 3. Results

On 1 April 2024, the systematic search came to an end. After screening 256 article titles, 30 abstracts were chosen for additional examination. Twenty articles were then examined in their entirety to see if they satisfied the inclusion requirements. Following an additional review, six articles were eliminated for the following reasons:

- Not a study in the field of AI application in implant planning (n = 3);
- Full text not available (n = 1);
- Missing information on AI technology (n = 2).

**Figure 1:**
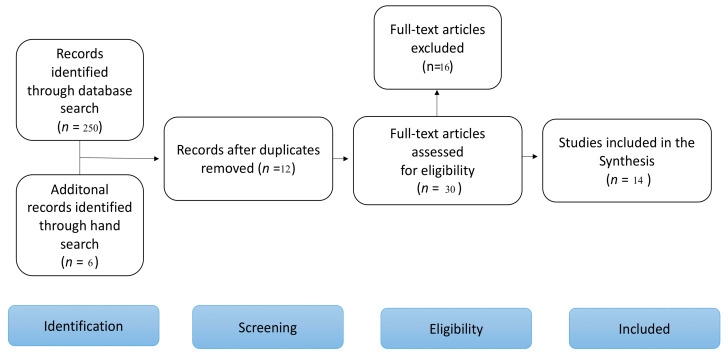
Search results, according to Page, M.J.; McKenzie, J.E.; Bossuyt, P.M.; Boutron, I.; Hoffmann, T.C.; Mulrow, C.D.; Shamseer, L.; Tetzlaff, J.M.; Akl, E.A.; Brennan, S.E.; et al. The PRISMA 2020 statement: An updated guideline for reporting systematic reviews. *BMJ* **2021**, *372*, n71. [24].

A total of 14 full-text papers were included for data extraction after two more articles that met the inclusion criteria were found through a manual search [9,10,11,12,13,14,15,16,17,18,19,20,21,22,23]. A visual summary with the numbers of studies screened, assessed for eligibility, and included in the review is presented in Figure 1 as a flow diagram. 

All data collected, Study Design, Aim of the Study, AI Application, Outcome or Conclusions, are collected in Table 3.

## 4. Discussion

Diagnostic procedures are a fundamental pillar in dental practice. An accurate diagnosis and detection of anatomic structures is the first step towards effective and personalized treatment [25,26]. In the field of oral implant rehabilitation, it is essential, to avoid intraoperative and postoperative complications, to plan the implant placement away from neural structures [27,28].

Therefore, accurate recognition of neural structures is of paramount importance. The first and most crucial step in planning mandibular implant treatment involves identifying the position of the inferior alveolar canal [29]. From the review we conducted, four scientific articles propose the identification of the mandibular nerve through the use of AI in implant diagnostic evaluation. All four studies describe the processes through which novel automated processes based on CNNs are developed to segment mandibular neural structures. Shuo Yang et al. [13] evaluated the recognition of the inferior alveolar nerve on two-dimensional panoramic images. The authors used machine learning on 1366 panoramic images, demonstrating that this method achieved high performance for IAC segmentation in panoramic images under different visibilities. Additionally, the authors demonstrated that the comparison between automated segmentation and manual annotation showed that the IAC position was highly consistent between the two segmentation approaches, with a matching degree close to 85%. Although the level of evidence of this study is high, considering the large number of datasets used and the detailed comparison with other methods, the diagnostic evaluation in implant planning using two-dimensional methods has many limitations. Therefore, despite utilizing innovative machine learning principles, it has limited utility in modern clinical implant planning [30]. Another study assessed the outcomes of a deep convolutional neural network on both two-dimensional and three-dimensional images obtained through machine learning from a dataset of 49,094 images acquired from CBCT scans. The authors conclude that while 3D U-Net demonstrated significantly superior results compared to 2D Net in automated canal nerve detection, deep learning will significantly enhance the efficiency of treatment planning. The other two studies evaluate the segmentation of neural structures on three-dimensional CBCT images. Oliveira-Santos N et al. [11] in their study mentioned the use of an AI-driven tool that provided accurate segmentation of the mandibular canal, even in the presence of anatomical variations such as an anterior loop. Thus, the currently validated dedicated AI tool may aid clinicians in automating the segmentation of neurovascular canals and their anatomical variations. It may significantly contribute to presurgical planning for dental implant placements, especially in the interforaminal region. Also, in the methodology described by Jindanil T. et al. [14] satisfactory results were achieved. Through machine learning of 200 CBCT scans, the authors concluded that the software autonomously recognizes the mandibular nerves. All four studies, despite the differences in computer methods, lead to the same conclusions, namely that automated segmentation of the mandibular canal on CBCT scans was proven to be accurate, time-efficient, and highly consistent, serving pre-surgical planning. Furthermore, the studies meet the criteria to be considered of a medium-high level of scientific evidence.

Two studies, Kurt Bayrakdar S. et al. [19] and Mangano F. et al. [22] instead evaluate models that more comprehensively detect and automatically segment various anatomical structures such as nerves, sinuses, bones, and missing teeth. Kurt Bayrakdar S. et al. [19] also include measurements related to bone thickness and height. Additionally, Mangano F. et al. [22] incorporate another crucial aspect into digital implant planning, namely the matching between CBCT segmentation and intraoral digital scanning, also achieved effectively in an automated manner. Although the first article describes a method performed on 75 CBCTs and we can consider the study to have a medium level of evidence, the method described by Mangano et al. [22] proposes a workflow used in a single case report. Therefore, further studies are needed to evaluate the level of evidence of the proposed method.

Among researchers in the field of machine learning, there is a debate about what should be the sufficient dataset size to create a proper learning curve for software. In all the retrospective studies we selected, we did not find homogeneity in the datasets used. Among the studies that used three-dimensional volumes of CBCT, the datasets varied from a minimum of 43 to a maximum of 200. Among the studies that evaluated the software based on two-dimensional images derived from panoramic X-rays or CBCT sections, the minimum dataset contained 316 images, while the study with the largest dataset had 16,272 images. Only the research conducted by Roongruangsilp P. et al. [18]. investigated the learning curve of the developed AI for dental implant planning, suggesting that regarding automated learning using images, the number of each image category used in AI development is positively related to AI interpretation. All authors agree that 50 images are the minimum image requirement. The primary stability achieved after implant insertion is crucial for promoting osseointegration [31]. This stability is influenced by the bone drilling protocol and the hardness of the bone [32]. A very interesting study explores the possibility of establishing a correct implant protocol supported by AI software. Takahiko S. et al. [9] demonstrated that artificial intelligence, after training on 960 images (taken from 2D sections of CBCT), can provide an effective method of predicting drilling protocols. A more recent study also assesses the clinical reliability of an AI-assisted implant planning software program with an in vitro model. Chen Z. et al. [23] obtained very satisfactory results and conclude by stating that the use of AI-supported software can effectively program the ideal implant position through self-learning. Additionally, they evaluate bone density, concluding that higher bone density led to increased implant deviations, emphasizing that it is one of the fundamental parameters to consider during implant planning. One article, instead, was selected concerning the anamnestic evaluations to be performed on the patient before implant surgery. Lyakhov et al. [21] introduced an artificial intelligence system designed to analyze patients’ statistical factors to predict the success of dental implant survival accurately. By utilizing a digitized database of clinical cases of osseointegration and an optimally designed neural network architecture tailored to these factors, a neural network system with a testing accuracy of 94.48% was achieved. In relation to this study, however, a higher level of evidence than Low could not be assigned, achieving a score of 4 in the overall star rating. In fact, as described by the authors, the proposed system cannot be used as a full-fledged tool for supporting medical decision-making. The study was conducted only as an additional diagnostic tool for a single dental implant.

The peri-implant site under preparation is commonly used by implantologists, especially in cases of very soft bone. The degree of under preparation is generally dictated by the clinician’s experience [33]. In implantology, the use of radiographic stents with landmarks helps to guarantee precise surgical planning [34]. This method involves the use of a radiographic stent, which is a personalized guide for the individual based on the patient’s radiographic data [35]. The radiographic stent contains radiographic landmarks, which are identifiable reference points on the panoramic radiograph or CBCT scan. Before or during the surgical procedure, the radiographic stent is placed on the patient’s panoramic radiograph or CBCT scan to ensure precise alignment of the landmarks with the patient’s anatomical structures. This allows the surgeon to accurately locate the position and angulation of dental implants based on pre-operative planning [36]. Alsomali D. et al. [20] developed a model that automatically localizes the position of radiographic stent markers in CBCT scans. However, the authors did not achieve reliable results. They clearly emphasize that the use of only axial images for training an AI program for the localization of gutta-percha markers is not sufficient to provide an accurate AI model performance. This result is consistent with the findings of the study by Hyunjung K.G. et al. [12], which highlights how the use of three-dimensional models showed significantly better results than 2D models. In the realm of medical science, diagnostic procedures are pivotal for the accurate detection of any anatomical structures and also to aid in accurate diagnosis and any subsequent treatment plans. Recently, the use of convolutional neural networks (CNNs) has proven to provide excellent performance in the field of 3D images analysis.

Firstly, the most crucial step in planning an implant in the maxillary region is identifying the anatomical structures and assessing the bone quality and quantity and any adjacent vital structures ex: maxillary sinus, nasal cavity, infraorbital nerve [36]. Hence, from the review we conducted two studies proposed the use of CNN in the detection of vital anatomical structures in the maxillary region through the use of AI in implant diagnostic evaluation. Both the studies describe the processes through which novel automated processes based on CNNs are developed to segment the maxillary sinus and bone. Nermin M. et al. [10] in their study developed and validated a novel automated CNN-based methodology for the segmentation of the maxillary sinus using CBCT images. The authors used in their study a dataset of 264 sinus images that were acquired from two CBCT devices. The authors also demonstrated that this method achieved a high performance for the detection of segmentation of the maxillary in CBCT images and proposed that the CNN model provided a time-efficient, precise, and consistent automatic segmentation which could allow an accurate generation of 3D models for diagnosis and virtual treatment planning to the comparison of manual segmentation of the maxillary sinus on CBCT images which the author proposed as time-consuming and dependent on the practitioner’s experience with high inter- and intra-observer variability. 

Another study assessed the outcomes of the use of CNNs in the maxillary region. Cavalcante F. R. et al. [16] evaluated a total of 141 CBCT scans and concluded that the AI-driven segmentation was 116 times faster than the manual segmentation; yet, although the use of manual segmentation showed a slightly better performance, the novel CNN-based tool also provided a highly accurate segmentation of the maxillary alveolar bone and its crestal contour consuming 116 times less than the manual approach. In another study, within its limitation relative to its small size a total of 43 CBCT datasets were evaluated. Adel Moufti M et al. [15] investigated the use of an artificial intelligence solution to auto-segmentation in the edentulous mandibular bone for implant planning. For this purpose, the author concluded that the use of segmentation of the edentulous spans on CBCT images was successfully conducted by machine learning with good accuracy compared to manual segmentation. It achieved a good accuracy (>90%) in segmenting unliteral cases, which represent the majority of patients with missing teeth. This automation of bone assessment on CBCT images has the potential to significantly reduce the time and associated cost of implant treatment.

Analyzing the potential legal implications of personal data protection in AI studies is also interesting. Health data are granted a high level of protection, such as within the European Union [Regulation (EU) 2016/679 of the European Parliament and of the Council of 27 April 2016 on the protection of natural persons with regard to the processing of personal data and on the free movement of such data and repealing Directive 95/46/EC (General Data Protection Regulation)], requiring an impact assessment of the potential use of the data prior to processing. It is thus essential to make the data to be used anonymous, not only by checking whether the patient can be identified in the document to be evaluated, but also by removing the metadata that may be present in a radiology examination that contains personal data [37,38]. This is essential when using external applications, as clinical data security may not be guaranteed. Ethics committees approving AI studies must also be aware of the potential leakage of personal data that may occur in this type of study and may even suggest additional security systems to researchers [39].

## 5. Conclusions

Although the number of studies indicates a growing interest in the application of AI in medicine, studies conducted on the use of modern artificial intelligence technologies for implant planning highlight the need for high-quality training data and the lack of standardization in protocols. 

However, even with different methods and datasets, automated recognition and segmentation methods of anatomical structures such as bones, nerves, teeth, and maxillary sinuses were proven to be accurate, time-efficient, and highly consistent. New studies are needed to evaluate implant success rates and drilling protocol assessments. The use of these methods conducted by machine learning can significantly contribute to efficient treatment planning, and the development of larger datasets can further enhance AI application performance. 

## Figures and Tables

**Table 1:** Search strategy.

Focused Question	What Are the Current Uses of AI in Implant Planning Described in the Literature?
**Search Population Strategy**	Scientific articles on the use of AI in implant planning
**Intervention or Exposure**	Electronic literature searches: #1 ((Implant Planning [Mesh] OR (implantology) OR (implant treatment plan)) Diagnostic model based on applied AI algorithms #2: ((Artificial Intelligence [Mesh] OR (AI) OR (machine learning) OR (deep learning))
**Comparison**	Traditional methods of implant planning
**Outcome**	Applications or diagnostic performance of the proposed AI model.
**Search combination**	#1 AND #2
**Database search**	PubMed, Scopus, Web of Science database
**Electronic journals**	Journal of Prosthodontic Research, Journal of Prosthetic Dentistry, Clinical Oral Implants Research, International Journal of Oral Maxillofacial Implants, Clinical Implant Dentistry and Related Research, Implant Dentistry, Journal of Implantology
**Selection criteria**	Studies at all levels of evidence, except expert opinion;
**Inclusion criteria**	Articles published in English; Articles published in the last 5 years.
**Exclusion criteria**	Review articles, letter to editors Animal studies; Multiple publications on the same patient population; Full text not available/accessible.

**Table 2:** Presentation of risk of bias evaluation for included studies (Newcastle–Ottawa Scale). A star system was implemented to allow a semi-quantitative assessment of study quality. The NOS ranges from zero to nine stars. We classified studies as high-quality if they achieved seven or more stars, medium-quality if they received four to six stars, and poor-quality if they had fewer than four stars.

	Selection	Comparability	Outcome	Overall Star Rating
	(Max. 4 Stars)	(Max. 2 Stars)	(Max. 4 Stars)	
Takahiko S. et al. (2023) [9]	***	*	***	7
Nermin M. et al. (2022) [10]	***	**	***	8
Oliveira-S N. et al. (2023) [11]	***	**	**	7
Hyunjung K.G. et al. (2023) [12]	***	**	***	8
Shuo Yang. et al. (2023) [13]	**	**	**	6
Jindanil T. et al. (2023) [14]	**	**	**	6
Adel Moufti M et al. (2023) [15]	**	**	***	7
Cavalcante F. R. et al. (2023) [16]	***	**	*	6
VinayahalingamS. et al. (2023) [17]	**	*	***	6
Roongruangsilp P. et al. (2021) [18]	**	*	**	5
Kurt Bayrakdar S. et al. (2021) [19]	**	*	***	6
Alsomali D. et al. (2022) [20]	**	**	*	5
Lyakhov P.A. et al. (2022) [21]	**	*	*	4
Mangano F. et al. (2023) [22]	-	-	-	-
Chen Z. et al. (2024) [23]	-	-	-	-

**Table 3:** Characteristics and outcomes of the studies included.

First Author (Year) Country	Study Design	n Datasets	Training/Validation Datasets	Test Datasets	Aim of the Study	AI Application	Outcome or Conclusions
Takahiko S. et al. (2023). Japan [9]	Retrospective study	1200 images (20 slices of 60 CBCT)	960 images, 80%	240 images, 20%	determination of an appropriate implant drilling protocol from CBCT scan	Keras library in Python. Adam optimizer was used to train the LeNet-5-based model.	Effective method of predicting drilling protocols from CBCT images before surgery
Cavalcante F. R. et al. (2023). Brazil [16]	Retrospective study	141 CBCT	99/12	22	Develop and assess the performance of a novel tool for automated three-dimensional (3D) maxillary alveolar bone segmentation on CBCT images.	The CNN models were developed in PyTorch	Although the manual segmentation showed slightly better performance, the novel CNN-based tool also provided a highly accurate segmentation of the maxillary alveolar bone
Adel Moufti M et al. (2023). United Arab Emirates [15]	Retrospective study	43 CBCT	33	10	Develop a solution to identify and delineate edentulous alveolar bone on CBCT	U-Net architecture CNN model	Segmentation of the edentulous spans on CBCT images was successfully conducted by machine learning with good accuracy compared to manual segmentation.
Nermin M. et al. (2022). Belgium [10]	Retrospective study	132 CBCT	83/19	30	Develop a novel automated CNN-based methodology for the segmentation of maxillary sinus on CBCT images	3D U-Net architecture CNN model	Promising performance in relation to time, accuracy and consistency
Kurt Bayrakdar S. et al. (2021). Turkey [19]	Retrospective study	75 CBCT, 508 regions	-	-	Evaluate an AI system in implant planning using CBCT. Evaluate canal/sinus/fossa, missing tooth detection, bone height measurements and bone thickness measurements	3D U-Net, CNN model	The success of the present study in the detection of sinus/mandibular canal and missing teeth and the measurements it offers in implant planning reinforces this possibility.
Oliveira-S N. et al. (2023). Brazil [11]	Retrospective study	220 CBCT	166/27	27	Train and validate a dedicated cloud-based AI-driven tool to allow accurate and timely segmentation of the mandibular canal and its anterior loop on CBCT scans	3D U-Net architecture CNN model	Contribute to presurgical planning for dental implant placement, especially in the interforaminal region
Hyunjung K.G. et al. (2023). Korea [12]	Retrospective study	102 CBCT	49,094 images/9818 images	9818 images	Valuate the automatic mandibular canal detection using a deep convolutional neural network	2D and 3D U-Net and 2D SegNet (CNN model)	Though 3D U-Net showed significantly better results than 2D Net in automated canal nerve detection. deep learning will contribute significantly to efficient treatment planning
Shuo Yang. et al. (2023). China [13]	Retrospective study	1366 2D panoramic images	1000 panoramic	336 panoramic	Evaluate the performance of automatic segmentation of inferior alveolar canal in panoramic images	EfficientUnet, CNN model	This method achieved high performance for IAC segmentation in panoramic images under different visibilities
Jindanil T. et al. (2023). Belgium, Brazil [14]	Retrospective study	200 CBCT	160/20	20	Develop and validate a novel tool for automated segmentation of mandibular incisive canal on CBCT scans	CNN model used on 3D U-net architecture	Automated segmentation of mandibular incisive canal on CBCT scans was proofed to be accurate, time efficient, and highly consistent, serving pre-surgical planning.
Vinayahalingam S. et al. (2023). Netherlands [17]	Retrospective study	1750 casts scans	1400	350	Develop an automated teeth segmentation and labeling system using deep learning	U-Net architecture CNN model	Promising foundation for time-effective and observer-independent teeth segmentation and labeling
Roongruangsilp P. et al. (2021). Thailand [18]	Retrospective study	316 images obtained from184 CBCT	300 images	16	Investigate the learning curve of the developed AI for dental implant planning in the posterior maxillary region	R-CNN algorithm	The number of each image category used in AI development is positively related to the AI interpretation. Fifty images are the minimum image requirement for over 70% positive prediction.
Alsomali D. et al. (2022). Saudi Arabia [20]	Retrospective study	34 CBCT, 16,272 axial images	90.2%/9.8%	4 cases	Develop a model that automatically localizes the position of radiographic stent markers in CBCT	R-CNN	Use of only axial images for training an AI program for localization of GP markers is not enough to give an accurate AI model performance.
Lyakhov P.A. et al. (2022). Russia [21]	Case Studies	1626 cases.	91.64% successful cases, 8.36% rejection cases	-	Propose a system for analyzing various patient statistics to predict the success of single implant survival	CNN architecture	A promising direction for further research is the development of a medical decision support system based on the technology for generating recommendations to reduce the risk of complications
Mangano F. et al. (2023) Italy [22]	Case report	1 case	-	-	Present a novel protocol for planning of dental implant	CNN architecture	Effective automatic alignment of digital intraoral scan and CBCT models, with CBCT segmentation
Chen Z. et al. (2024). China [23]	In vitro study	10 cases	-	-	Determine the clinical reliability of an AI-assisted implant planning software program with an in vitro model	CNN architecture	AI implant planning software program could design the ideal implant position through self-learning. Higher bone density led to increased implant deviations.

## Data Availability

The raw data supporting the conclusions of this article will be made available by the authors on request.
